# Exacerbation of symptoms, nocturnal acid reflux, and impaired autonomic function are associated with sleep disturbance in gastroesophageal reflux disease patients

**DOI:** 10.3389/fmed.2024.1438698

**Published:** 2024-08-21

**Authors:** Yizhou Huang, Jie Liu, Linsheng Xu, Wu Qi, Jie Dai, Bo Wang, Jiashuang Tian, Xin Fu, Yue Yu

**Affiliations:** ^1^Department of Gastroenterology, The Anqing 116 Hospital, China RongTong Medical Healthcare Group Co. Ltd., Anqing, Anhui, China; ^2^Department of Gastroenterology, The First Affiliated Hospital of USTC, Division of Life Sciences and Medicine, University of Science and Technology of China, Hefei, Anhui, China

**Keywords:** gastroesophageal reflux disease, sleep disturbance, autonomic dysfunction, anxiety, depression

## Abstract

**Background and aim:**

Gastroesophageal reflux disease (GERD) patients often report sleep disturbance (SD); however, the relationship between GERD and SD is unknown. This study investigated whether SD affects symptoms, acid reflux, and autonomic function in GERD patients.

**Methods:**

A total of 257 subjects (126 patients with SD and 99 patients without SD) participated in this survey from January 2020 to August 2022. Participants were required to complete questionnaires including the GERD impact scale (GIS), Hamilton Anxiety Scale (HAMA), and Hamilton Depression Scale (HAMD). Esophageal mucosal injury, acid exposure, peristaltic function, and autonomic function were assessed by upper endoscopy, high-resolution esophageal manometry (HRAM), 24-h multichannel intraluminal impedance with pH (24 h-MII-pH), and electrocardiography (ECG).

**Results:**

Gastroesophageal reflux disease patients with SD experienced a higher frequency of prolonged reflux (*p* < 0.001), longest reflux event (*p* < 0.001), acid exposure time (*p* < 0.001) during the recumbent period, and a higher incidence of erosive esophagitis (EE) (59.5 vs. 45.5%, *p* = 0.036) than those without SD. Pearson’s correlation analysis showed that SD was positively correlated with GIS (*r* = 0.725, *p* < 0.001), HAMA (*r* = 0.680, *p* < 0.001), and HAMD (*r* = 0.323, *p* < 0.001) scores, and negatively correlated with parasympathetic or vagal nerve activity (*r* = −0.770, *p* < 0.001).

**Conclusion:**

Gastroesophageal reflux disease patients with SD experience more severe reflux symptoms and nocturnal acid reflux, which may be related to autonomic dysfunction.

## 1 Introduction

Gastroesophageal reflux disease (GERD) is a chronic disease caused by the reflux of gastroduodenal contents due to various causes, leading to uncomfortable symptoms and complications (1). 13% of the world’s population is estimated to experience GERD symptoms at least once per month (2). Proton-pump inhibitors (PPIs) remain the mainstay of treatment for GERD, but 40% of patients reportedly experience no significant relief of reflux symptoms after 8 weeks of full dosage and full course of acid suppressant (3). Interestingly, Schey et al. showed that sleep deficiency (SD) could induce esophageal hypersensitivity in GERD patients and may be one of the reasons why reflux symptoms are difficult to control in some patients (4).

Sleep disturbance (SD) usually refers to a subjective experience whereby the patient is dissatisfied with the duration and/or quality of sleep and affects daytime social functioning (5). Indeed, good sleep hygiene is an important component of overall health and quality of life. People that get less than 5–6 h of sleep per night are classified as sleep deprived. Over the past century, the average night sleep has decreased by 2 h (6, 7). SD can lead to many health-related diseases such as obesity, diabetes, cardiovascular disease, functional gastrointestinal disorders (FGIDs), reduced immune response, impaired cognition, anxiety and depression, poor quality of life, and increased all-cause mortality (8). SD is also associated with disorders of gut-brain interaction (DGBI), such as irritable bowel syndrome (IBS) and functional dyspepsia (FD), and often results in various gastrointestinal symptoms, including abdominal pain, bloating, and belching (5, 7). Current evidence suggests increased risk of multiple gastrointestinal complaints and reduced quality of life are associated with SD (9). A recent study emphasized the importance of properly coordinating the sleep/wake cycle with the body’s circadian cycle for achieving healthy sleep. Therefore sleep, wake, and meal times should match the circadian cycle as closely as possible, to obtain optimal sleep and gut-related health (7).

Proton-pump inhibitors continue to be the preferred pharmacological treatment for gastroesophageal reflux disease (GERD). However, numerous studies have highlighted potential adverse events associated with their use, thereby casting doubt on the safety of prolonged administration and heightening concerns regarding the overprescription of these medications. Additionally, recent evidence has surfaced concerning the viability of surgical and endoscopic alternatives for managing this condition. Meanwhile, mineral waters, especially those based on bicarbonate-magnesium, were also validated for the management of GERD (10, 11).

It has been established that GERD is a kind of FGID often associated with SD. However, most studies have demonstrated the association between gastrointestinal symptoms and SD, while the relationship between SD and esophageal motility in GERD has been largely understudied. Therefore, this study aimed to investigate the effect of SD on esophageal motility and autonomic function in GERD (10).

## 2 Materials and methods

### 2.1 Subjects

Patients diagnosed with GERD based on diagnostic endoscopy and reflux monitoring at the Department of Gastroenterology, the First Affiliated Hospital of the University of Science and Technology of China (USTC) from January 2020 to August 2022 were enrolled in the study (1). The diagnosis of GERD was defined based on Lyon consensus 2.0 (12). In detail, the diagnostic criteria of included patients were: (1) LA grade B, C, and D esophagitis, biopsy proven Barrett’s esophagus and peptic stricture; (2) AET > 6.0%, >80 reflux episodes, and MNBI value <1,500 ohms; (3) borderline evidence with supportive adjunctive metrics according to the Lyon Consensus. The exclusion criteria consisted of: (1) patients with presence of other pathologies leading to altered sleep, such as heart, lung, liver, kidney diseases, or other serious comorbidities, (2) patients with any previously serious psychiatric comorbidity, such as HAMD>20; (4) patients who used psychotropic drugs or medication influencing sleep; (5) patients who had taken PPI 1 week prior to inclusion.

The study was approved by the Ethics Committee of the First Affiliated Hospital of USTC (Registration No: 2022-RE-296) and was performed in accordance with the Declaration of Helsinki. All participants gave written informed consent for enrollment in this study.

### 2.2 Study protocol

The patients included in this study were divided into two groups based on their Pittsburgh Sleep Quality Index (PSQI) scores: GERD patients with SD (PSQI scores ≥ 5) and GERD patients without SD (PSQI scores < 5). All participants were required to complete questionnaires, including PSQI, GERD impact scale (GIS), Hamilton Anxiety Scale (HAMA), and Hamilton Depression Scale (HAMD). Moreover, all participants underwent High-resolution esophageal manometry (HRAM), 24-h multichannel intraluminal impedance with pH (24 h-MII-pH), upper endoscopy, and electrocardiogram (ECG).

### 2.3 Sample size

The sample size was calculated, referencing a previous clinical study (13). As a case–control study, in the control group, the sleep disturbance (defined as PSQI) prevalence was 65.61% (332/506), while the adjusted OR value was 3.5. To achieve a power of 80%, a sample size of 62 patients in case and control group was necessary, considering a 20% dropout rate and an alpha level of 5%. Therefore, the sample size of this study is sufficient to demonstrate statistical differences.

### 2.4 Measurements

#### 2.4.1 Questionnaires

##### 2.4.1.1 Pittsburgh sleep quality index

The Pittsburgh Sleep Quality Index score is a standardized rating scale that captures the global sleep status in different populations, including the general population and individuals with psychiatric disorders. It identifies sleep disorders by assessing seven domains: subjective sleep quality, sleep latency, sleep duration, habitual sleep efficiency, sleep disturbance, sleep medication use, and daytime dysfunction. Each category is scored on a scale of 0–3, and these factors are added together to give a global score (maximum global score of 21). Higher scores indicate poorer sleep, with a global score above 5 considered “SD” (14–16).

##### 2.4.1.2 GERD impact scale

The GERD impact scale is a practical and easily administered instrument targeted for use in the primary care setting. The questionnaire includes nine items covering five symptom domains (chest pain, heartburn, acid reflux, epigastric pain, and hoarseness) and four questions on the quality of life (QOL). Their frequency of symptoms and poor quality of life in the past 1 month were assessed separately using a modified five-point Likert scale (0, never; 1, 1–2 times per month; 2, 1–2 times per week; 3, 3–4 times per week; 4, daily). The GIS scores range from 0 to a maximum of 36 (17).

##### 2.4.1.3 HAMA and HAMD scale

The Hamilton anxiety scale and Hamilton depression scale have been widely used to assess the mental state of patients (18, 19). The Hamilton anxiety scale contains 14 items; each assessed separately using a five-point Likert scale ranging from grade 0 to grade 4. A global HAMA score of >6 indicates “anxiety.” HAMD contains 17 items; each scored on a five-point Likert scale. A global HAMA score of >7 indicates “depression” (20, 21).

#### 2.4.2 Upper endoscopy

Upper endoscopy is the most widely used examination to assess the esophageal mucosa, especially the distal portion of the esophagus, during standard endoscopy to determine the presence of mucosal injury. The Los Angeles (LA) classification was used to assess the severity of erosive esophagitis (EE) as follows: Grade A (one or more esophageal mucosal breaks with a maximum diameter < 5 mm), Grade B (one or more esophageal mucosal breaks with a maximum diameter > 5 mm but no fused lesions), Grade C (esophageal mucosal breaks with fusion but <75% of the circumference of the esophagus), and Grade D (esophageal mucosal breaks with fusion to at least 75% of the circumference of the esophagus) (1, 22).

#### 2.4.3 High-resolution esophageal manometry

A water-perfused esophageal manometric catheter with 24 pressure sensors spaced at 1 cm intervals (MedKinetic, Ningbo, China) was used to assess the motility of the esophagus. All patients were taken off acid suppressants and prokinetic drugs for at least 1 week before HREM, and subjects were required to fast for at least 8 h before the test. During the examination, the HREM catheter was inserted through the patient’s unobstructed nasal cavity, and the patient was placed in a prone position after successful insertion. After recording the resting state pressure, the patient swallowed 5 mL of water 10 times every 30 s. The catheter was removed after manometry was completed (23).

#### 2.4.4 Multichannel intraluminal impedance with pH testing

A combined 24-h multichannel intraluminal impedance with a pH (pH-MII) testing system (Jinshan, Chongqing, China) and a 6MII-1 pH electrode catheter (JSIpS-1) were used to perform 24-h pH-impedance monitoring of the esophagus. After the position of the LES was determined by HREM, the monitoring catheter was inserted through the nasal cavity on one side of the patient and placed, ensuring that the pH electrode was located 5 cm above the upper edge of the LES. After fixing the catheter and the monitoring system was turned on, the patient was instructed to record his diet, postural changes and the onset of GERD symptoms during the monitoring period (24).

#### 2.4.5 Autonomic functions

The Heart Rate Variability (HRV) was documented by electrocardiogram (ECG) recording (ct-082, Hangzhou Baihui Electrocardiograms, China) and imported into HRV analysis software (Cardiotrak Holtersystem version: 1.2.0.0, Hangzhou Baihui Electrocardiograms, China). HRV frequency domain indicators include a low frequency (LF) band (LF power in the range of 0.04–0.15 Hz) and a high frequency (HF) band (HF power in the range of 0.15–0.40 Hz). Given that LF is an indicator of sympathetic activity, and HF is an indicator of parasympathetic or vagal activity, the LF/HF ratio can be used to assess sympathetic/parasympathetic balance (25).

### 2.5 Statistical methods

All statistical analyses were performed using SPSS version 22.0 software. Continuous variables were expressed as mean ± standard deviation (SD) and using independent samples t-test analysis. Categorical variables were expressed as rates and compared using the χ^2^ test. Correlations were assessed using Pearson’s correlation test. A *p* value <0.05 was statistically significant.

## 3 Results

### 3.1 Patient characteristics

**Figure 1:**
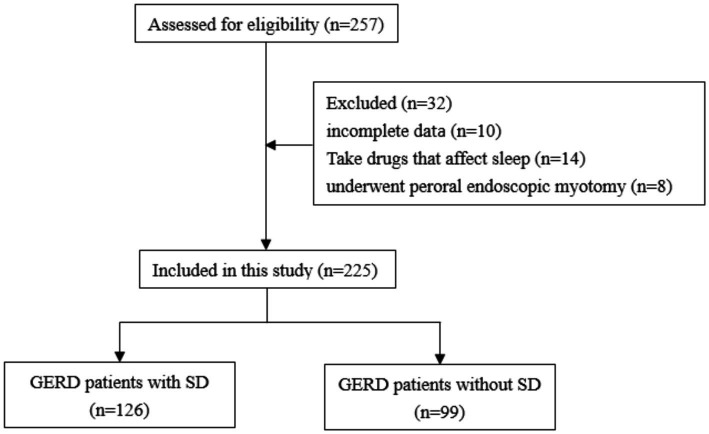
Flow chart of GERD patient recruitment in this research.

257 GERD patients initially participated in this study. 32 patients were excluded due to incomplete data (*n* = 10), use of drugs that affected sleep (*n* = 14), or history of peroral endoscopic myotomy (*n* = 8). Finally, 225 GERD patients were enrolled in this study (Figure 1) with a mean age of 45.60 ± 11.12 years (range 22–83 years) and separated into patients with (*n* = 126) or without (*n* = 99) SD. The detailed characteristics of included patients were shown in Table 1. A total of 12 patients with esophagitis A had pathologic pH-impedance (AET > 6%, reflux episodes >80, and MNBI <1,500 ohms) as evidence with supportive adjunctive metrics according to the Lyon Consensus. A were the sex ratio, mean age and body mass index did not differ significantly between both patient groups (Table 2).

**Table 1:** The details of included subjects.

Variables category	Included GERD patients (*N* = 225)
PPI responder (*n*, %)	178 (79.1%)
PPI non-responder (*n*, %)	47 (20.9%)
AET > 6% (*n*, %)	203 (90.2%)
AET between 4 and 6% (*n*, %)	22 (9.8%)
MNBI <1,500 ohms (*n*, %)	198 (88.0%)
MNBI >1,500 ohms (*n*, %)	27 (22.0%)
Total reflux episodes >80 (*n*, %)	201 (89.3%)
Total reflux episodes <80 (*n*, %)	24 (10.7%)
Esophagitis A (*n*, %)	12
Esophagitis B (*n*, %)	43
Esophagitis C (*n*, %)	52
Esophagitis D (*n*, %)	12

**Table 2:** Demographics in GERD patients with SD and without SD.

Variables category	GERD with SD (*n* = 126)	GERD without SD (*n* = 99)	*t*	*p* value
Age (years)	45.71 ± 11.49	45.45 ± 10.69	0.174	0.862
Male, *n* (%)	53 (42.1%)	50 (50.5%)	1.592	0.207
BMI (kg/m^2^)	22.45 ± 3.39	23.27 ± 3.73	−1.732	0.085
Duration of GERD (months)	9.85 ± 1.55	9.67 ± 1.56	0.875	0.383
PSQI score	11.50 ± 3.79	2.90 ± 1.28	23.801	<0.001

GERD, Gastroesophageal reflux disease; SD, Sleep disturbance; BMI, Body mass index; and PSQI, Pittsburgh sleep quality index.

### 3.2 Comparison of the GERD impact scale, anxiety, and depression score between both groups

Comparison of both groups showed that the frequency of heartburn (3.63 ± 1.00 vs. 3.24 ± 0.72, *p* = 0.001), acid regurgitation (3.66 ± 0.95 vs. 3.25 ± 0.72, *p* < 0.001), and hoarseness (2.70 ± 0.58 vs. 1.26 ± 0.60, *p* < 0.001) was significantly higher in GERD patients with SD, while the frequency of chest pain (*p* = 0.461) and epigastric pain (*p* = 0.329) was comparable. The frequency of sleep disturbance (3.75 ± 1.05 vs. 0.90 ± 0.53, *p* < 0.001), limitation on daily activities (3.28 ± 0.88 vs. 0.87 ± 0.49, *p* < 0.001), and use of additional medication (2.77 ± 0.52 vs. 0.74 ± 0.55, *p* < 0.001) were higher in GERD patients with SD. There was no statistical difference regarding eating problems (*p* = 0.895) among these two groups. The total GIS score (23.32 ± 3.76 vs. 13.82 ± 2.00, *p* < 0.001) was significantly higher in GERD patients with SD (Table 3). Meanwhile, GERD patients with SD had higher HAMA (9.54 ± 3.13 vs. 5.56 ± 2.69, *p* < 0.001) and HAMD (8.20 ± 3.20 vs. 5.97 ± 2.67, *p* < 0.001) scores compared with GERD patients without SD (Table 4).

**Table 3:** The GIS score in GERD patients with SD and without SD.

GIS score	GERD with SD (*n* = 126)	GERD without SD (*n* = 99)	*t*	*p* value
Chest pain	1.07 ± 0.62	1.01 ± 0.61	0.738	0.461
Heartburn	3.63 ± 1.00	3.24 ± 0.72	3.295	0.001
Acid regurgitation	3.66 ± 0.95	3.25 ± 0.72	3.538	<0.001
Epigastric pain	1.48 ± 0.59	1.57 ± 0.66	−0.979	0.329
Hoarseness	2.70 ± 0.58	1.26 ± 0.60	18.052	<0.001
Sleep disturbance	3.75 ± 1.05	0.90 ± 0.53	24.761	<0.001
Eating problems	0.97 ± 0.67	0.98 ± 0.64	−0.132	0.895
Limitation on daily activities	3.28 ± 0.88	0.87 ± 0.49	24.395	<0.001
Use of additional medication	2.77 ± 0.52	0.74 ± 0.55	28.360	<0.001
Total score	23.32 ± 3.76	13.82 ± 2.00	24.310	<0.001

GIS, Gastroesophageal reflux disease impact scale; GERD, Gastroesophageal reflux disease; SD, Sleep disturbance.

**Table 4:** The HAMA and HAMD scores in GERD patients with SD and without SD.

	GERD with SD (*n* = 126)	GERD without SD (*n* = 99)	*t*	*p* value
HAMA	9.54 ± 3.13	5.56 ± 2.69	10.263	<0.001
HAMD	8.20 ± 3.20	5.97 ± 2.67	5.686	<0.001

GERD, Gastroesophageal reflux disease; SD, Sleep disturbance; HAMA, Hamilton anxiety scale; and HAMD, Hamilton depression scale.

### 3.3 Comparison of the severity of erosive esophagitis between the two groups

Erosive esophagitis was found more in GERD patients with SD than those without SD (59.5 vs. 45.5%, *p* = 0.036). According to the LA classification, the proportion of grade A (*p* = 0.667) and grade D (*p* = 0.867) was comparable between the two groups. The proportion of grade B GERD patients with SD was 14.3%, lower than GERD patients without SD (*p* = 0.038), while the proportion of grade C was 34.9%, higher than GERD patients without SD (*p* < 0.001) (Table 5).

**Table 5:** The severity of erosive esophagitis in GERD patients with SD and without SD.

EE (*n* [%])	GERD with SD (*n* = 126)	GERD with SD and normal HAMA/HAMD (*n* = 11)	GERD without SD (*n* = 99)	*χ* ^2^	*p* value
None	51(40.5)	2 (18.2)	54 (54.5)	4.409	0.036
LA-A	6 (4.8)	2(18.2)	6 (6.1)	0.185	0.667
LA-B	18 (14.3)	3(27.3)	25 (25.3)	4.313	0.038
LA-C	44 (34.9)	3(27.3)	8 (8.1)	22.475	<0.001
LA-D	7 (5.6)	1(9.1)	5 (5.1)	0.028	0.867

EE, Erosive esophagitis; GERD, Gastroesophageal reflux disease; SD, Sleep disturbance; LA, Los Angeles classification.*χ*^2^ was calculated between GERD with SD and without SD.

### 3.4 Comparison of the high-resolution esophageal manometry between the two groups

The Lower Esophageal Sphincter resting Pressure (LESP) (*p* = 0.290) and Lower Esophageal Sphincter Residual Pressure (LESRP) (*p* = 0.349) were lower in GERD patients with SD than without SD; however, there was no significant difference between the two groups. Meanwhile, the Upper Esophageal Sphincter resting Pressure (UESP) (*p* = 0.316), Upper Esophageal Sphincter Residual Pressure (UESRP) (*p* = 0.188), Contractile Front Velocity (CFV) (*p* = 0.474) and Distal Contractile Integral (DCI) (*p* = 0.105) were not significantly different between the two groups (Table 6).

**Table 6:** The esophageal function in GERD patients with SD and without SD.

HREM data	GERD with SD (*n* = 126)	GERD with SD and normal HAMA/HAMD (*n* = 11)	GERD without SD (*n* = 99)	*t*	*p* value
UESP (mmHg)	48.67 ± 13.37	46.28 ± 9.26	50.52 ± 14.19	−1.005	0.316
UESRP (mmHg)	8.00 ± 1.91	8.14 ± 1.18	7.67 ± 1.86	1.321	0.188
LESP (mmHg)	8.09 ± 1.96	8.17 ± 1.33	8.36 ± 1.70	−1.061	0.290
LESRP (mmHg)	4.97 ± 1.29	5.04 ± 1.14	5.13 ± 1.26	−0.939	0.349
CFV (cm/s)	4.08 ± 1.01	4.11 ± 0.96	3.98 ± 1.02	0.717	0.474
DCI (mmHg·s·cm)	2236.19 ± 711.57	2295.27 ± 639.41	2394.14 ± 734.99	−1.629	0.105

HREM, High-resolution esophageal manometry; GERD, Gastroesophageal reflux disease; SD, Sleep disturbance; UESP, Upper esophageal sphincter resting pressure; UESRP, Upper esophageal sphincter residual pressure; LESP, Lower esophageal sphincter resting pressure; LESRP, Lower esophageal sphincter residual pressure; CFV, Contractile front velocity; DCI, Distal contractile integral.*t* value was calculated between GERD with SD and without SD.

### 3.5 Comparison of the 24 h-MII-pH testing between the two groups

The frequency of prolonged reflux during the recumbent period was significantly greater in GERD patients with SD than without SD (2.74 ± 1.07 vs. 2.35 ± 0.76, *p* < 0.001), while no significant differences were found for the overall and upright periods in both groups (*p* = 0.114 and *p* = 0.653, respectively). Although there was no difference in longest reflux time during the overall and upright periods in both groups (*p* = 0.664 and *p* = 0.300, respectively), GERD patients with SD experienced a significant increase in the longest reflux time compared with those without SD during the recumbent period (3.40 ± 1.76 vs. 2.93 ± 1.29, *p* < 0.001). Moreover, AET was significantly greater in GERD patients with SD than without SD in the recumbent period (3.08 ± 1.16 vs. 2.62 ± 1.12, *p* < 0.001) (Table 7).

**Table 7:** The 24 h-MII-pH testing data in GERD patients with SD and without SD.

24 h-MII-pH data	GERD with SD (*n* = 126)	GERD with SD and normal HAMA/HAMD (*n* = 11)	GERD without SD (*n* = 99)	*t*	*p* value
Number of prolonged reflux (*n*, >5 min)					
Total	4.75 ± 1.46	4.68 ± 1.30	4.44 ± 1.35	1.587	0.114
Upright	2.01 ± 1.47	1.99 ± 1.27	2.09 ± 1.24	−0.451	0.653
Recumbent	2.74 ± 1.07	2.77 ± 0.98^*^	2.35 ± 0.76	3.153	<0.001
Longest reflux event (minutes)					
Total	7.66 ± 1.58	7.58 ± 1.49	7.57 ± 1.55	0.435	0.664
Upright	6.72 ± 2.84	6.70 ± 2.79	7.08 ± 2.40	−1.038	0.300
Recumbent	3.40 ± 1.76	3.38 ± 1.44^*^	2.93 ± 1.29	2.299	<0.001
AET (%)					
Total	7.57 ± 2.06	7.49 ± 2.01	7.34 ± 2.07	0.827	0.409
Upright	5.75 ± 1.31	5.71 ± 1.28	5.58 ± 1.44	0.920	0.358
Recumbent	3.08 ± 1.16	3.11 ± 1.15^*^	2.62 ± 1.12	2.982	<0.001

24 h-MII-pH, 24-h multichannel intraluminal impedance with pH; GERD, Gastroesophageal reflux disease; SD, Sleep disturbance; AET, Acid exposure time. *t* value was calculated between GERD with SD and without SD.^*^p < 0.05, calculated between GERD with SD and normal HAMA/HAMD and GERD without SD groups.

### 3.6 Comparison of the autonomic functions between the two groups

Gastroesophageal reflux disease patients with SD had a higher LF/HF ratio than in patients without SD (1.19 ± 0.24 vs. 0.89 ± 0.12, *p* < 0.001), and had a lower HF/LF ratio than in patients without SD (0.88 ± 0.19 vs. 1.15 ± 0.16, *p* < 0.001) (Table 8).

**Table 8:** The autonomic functions in GERD patients with SD and without SD.

	GERD with SD (*n* = 126)	GERD with SD and normal HAMA/HAMD (*n* = 11)	GERD without SD (*n* = 99)	*t*	*p* value
HF/LF	0.88 ± 0.19	0.90 ± 0.17^*^	1.15 ± 0.16	−11.823	<0.001
LF/HF	1.19 ± 0.24	1.22 ± 0.20^*^	0.89 ± 0.12	12.239	<0.001

GERD, Gastroesophageal reflux disease; SD, Sleep disturbance; LF, Low frequency; HF, High frequency.*t* value was calculated between GERD with SD and without SD.^*^p < 0.05, calculated between GERD with SD and normal HAMA/HAMD and GERD without SD groups.

### 3.7 Correlations between Pittsburgh sleep quality index and GERD impact scale, anxiety score, depression score, and autonomic functions

**Figure 2:**
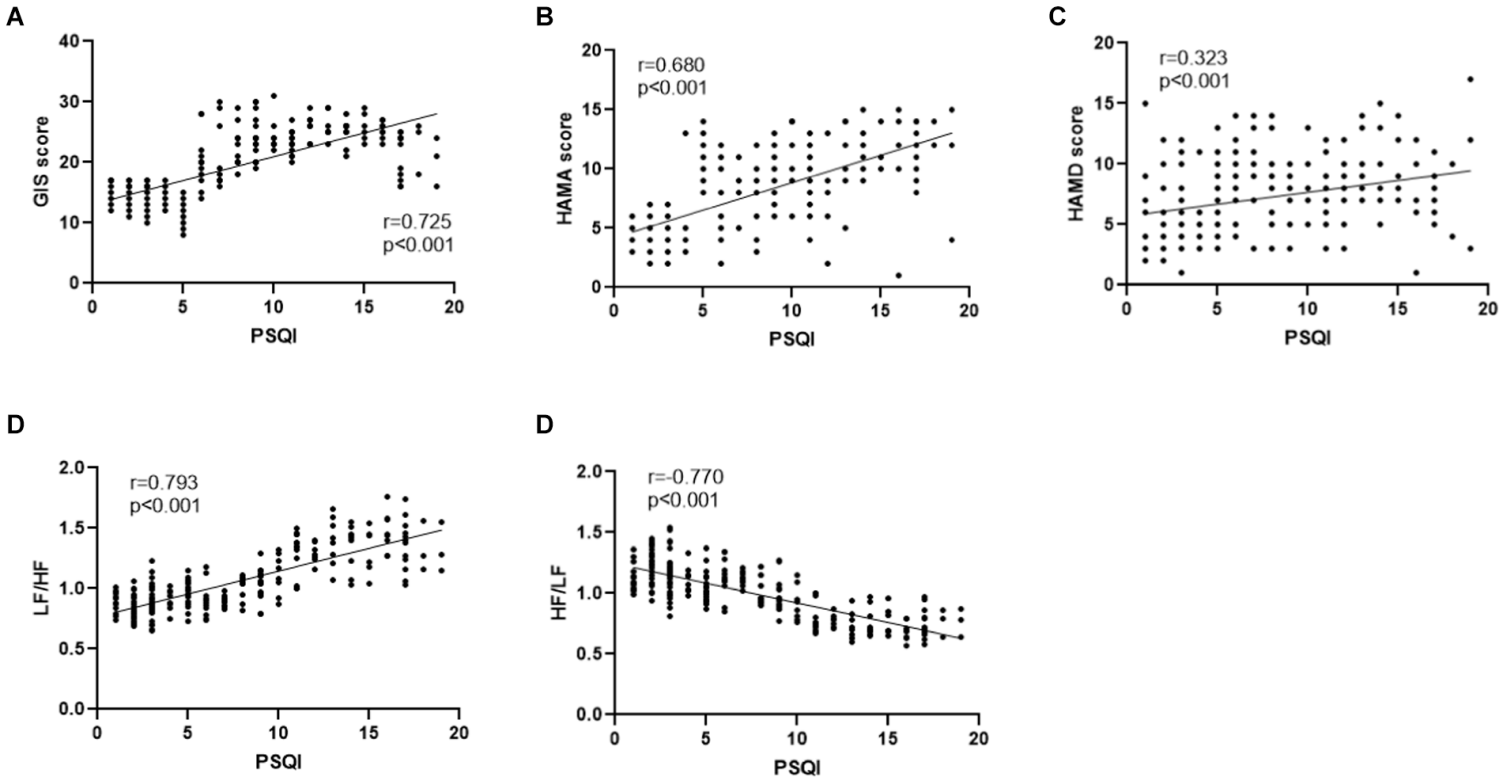
**(A)** PSQI was positively correlated with GIS in GERD patients (*r* = 0.725, *p* < 0.001). **(B)** PSQI was positively correlated with HAMA score in GERD patients (*r* = 0.680, *p* < 0.001). **(C)** PSQI was positively correlated with HAMD score in GERD patients (*r* = 0.323, *p* < 0.001). **(D)** PSQI was positively correlated with LF/HF in GERD patients (*r* = 0.793, *p* < 0.001). **(E)** PSQI was negatively correlated with HF/LF in GERD patients (*r* = −0.770, *p* < 0.001).

Pittsburgh Sleep Quality Index was positively correlated with the GIS score (*r* = 0.725, *p* < 0.001), HAMA score (*r* = 0.680, *p* < 0.001), HAMD score (*r* = 0.323, *p* < 0.001), and LF/HF (*r* = 0.793, *p* < 0.001) in GERD patients. However, PSQI was negatively correlated with HF/LF (*r* = −0.770, *p* < 0.001) in GERD patients (Figures 2A–E).

## 4 Discussion

This study revealed the effects of SD on reflux symptoms in GERD patients and explored the underlying autonomic mechanisms through spectral analysis of heart rate variability. Our data showed that GERD patients with SD experience a higher incidence of esophageal symptoms (especially heartburn, acid regurgitation and hoarseness) and nocturnal acid reflux than those without SD, with decreased quality of life and increased anxiety and depression. Notably, we found that GERD patients with SD had a higher incidence of erosive esophagitis with more severe damage to the esophageal mucosa. Meanwhile, SD increased sympathetic activity while suppressing parasympathetic or vagal activity. Pearson correlation analysis showed that SD was positively correlated with GIS scores, HAMA scores, HAMD scores, and sympathetic activity and negatively correlated with parasympathetic or vagal activity. Notably, subgroup analysis on patients with increased PSQI but with normal HAMA and HAMD was also conducted. We found that GERD patients with SD and normal HAMA/ HAMD also had a higher incidence of erosive esophagitis with more severe damage to the esophageal mucosa. Meanwhile, GERD patients with SD and normal HAMA/HAMD also had increased sympathetic activity while decreased parasympathetic or vagal activity, which bring valuable information on the influence of sleep on GERD.

The sample size can mainly provide medium-to-large effect sizes, and we found that the sample size of this study is sufficient to demonstrate statistical differences although the statistical power is not very high. The association between SD and GERD has long been reported in the literature (26). A large cross-sectional study documented that heartburn and regurgitation were almost twice as likely in patients with SD as in those without SD (27). In an Australian community-based study where 1,612 men completed a GERD symptom questionnaire, it was found that GERD symptom severity was significantly positively associated with self-reported poor quality of sleep (28). This association was further supported by a recent study investigating patients with various gastrointestinal disorders in a tertiary gastrointestinal clinic (29). Meanwhile, PSQI is widely acknowledged as a major tool for assessing sleep quality. We found that PSQI scores in GERD patients with SD were positively correlated with the severity of GERD symptoms. SD is widely thought to be a transdiagnostic symptom for many mental disorders, being most closely related to depression and anxiety (30, 31). SD was likewise found to influence anxiety and depressive states and positively correlate with those states in patients in our study.

Dickman et al. used questionnaires to evaluate SD in GERD patients and found that erosive reflux disease subjects woke up with reflux symptoms more frequently than those with non-erosive reflux disease (32). A Japanese study found that the rate of SD in patients with GERD was about one-third and that patients with EE had significantly shorter sleep times (33), given that nocturnal acid reflux might interfere with normal sleep by inducing conscious awakening during sleep. Recumbent acid reflux is associated with GERD complications such as increased EE (34). Our study found that EE was more frequent and mucosal damage was more severe in GERD patients with SD, consistent with the literature (32). Previous studies substantiated that GERD patients with SD experience reduced esophageal mechanosensitivity in generating distension-induced secondary peristalsis and a reduced successful peristaltic response (35). However, we did not find a significant correlation between SD, esophageal sphincter pressure, and esophageal motility in GERD patients. Interestingly, a Swedish study found that patients with persistent nocturnal GERD symptoms experienced a significantly higher prevalence of daytime sleepiness than those without nocturnal GERD symptoms. Moreover, benzodiazepines could affect the basal lower esophageal sphincter pressure and transient lower esophageal sphincter relaxation rate (36).

It has been established that GERD can affect the sleep–wake cycle and lead to SD. Conversely, SD has a strong impact on GERD. Therefore, sleep and GERD are bidirectionally associated (37). A previous study reported that feedback loops in the gut-brain axis could influence the regulation of circadian rhythms via mechanisms, including immune activation and intestinal endocrine signaling (38). 24 h-MII-pH showed an association between impaired sleep quality and nocturnal acid reflux events and longer reflux duration, consistent with the findings of Dickman et al. (32). This phenomenon may be attributed to decreased esophageal peristalsis and prolonged esophageal acid clearance at night. Accordingly, GERD patients have nocturnal reflux symptoms resulting in an increased risk of SD (39). A study reported that SD increased esophageal acid exposure in GERD patients, resulting in abnormal pH tests in nearly half of healthy individuals (40). Recent studies have found that the left lateral position significantly reduced the duration of nocturnal esophageal acid exposure time and accelerated esophageal acid clearance compared to the supine and right lateral positions (41). Meanwhile, the position of the head during sleep may also cause heartburn and affect sleep quality. One study investigated the effect of head position on reflux symptoms and sleep quality by gradually elevating the head over 7 days, and the results indicated that elevating the head reduced acid exposure and acid clearance time of the esophagus in the recumbent period (42). Meanwhile, our previous study (43) found that SD is associated with worse constipation related symptoms, and impaired autonomic function in functional constipation patients.

Motor regulation of the gastrointestinal tract is influenced by the enteric nervous system located within the intestinal wall and combined with extrinsic autonomic innervation. The vagus nerve has a major influence on esophageal peristalsis and LES function. Moreover, sympathetic excitation can reportedly stimulate the esophageal sphincter tone and reduce motility (44). HRV provides a non-invasive method of assessing autonomic function (25). A previous study found that esophageal acid exposure is usually associated with decreased autonomic tone (45). Meanwhile, Wang et al. (46) found that poor sleep resulted in higher LF and a lower HF/LF ratio, reflecting increased sympathetic and decreased parasympathetic activity. Notably, in a previous study, we documented that the imbalance between sympathetic and parasympathetic nerves plays an important role in refractory GERD (23). The current study substantiated that SD affects the balance between parasympathetic and sympathetic, thus significantly decreasing parasympathetic activity.

## 5 Limitations

Our study had some limitations that need to be addressed. First, although patients with comorbid psychiatric disorders were excluded, it is well-established that there is a definite relationship between sleep problems and mental status, which may affect our results to a certain extent. Besides, we defined SD based on the results of the PSQI questionnaire but did not assess objective sleep parameters, such as polysomnography, which may also affect our results. Finally, participants in this study underwent 24 h-MII-pH, which proved to be less sensitive and led to physical discomfort compared to wireless pH monitoring (47).

## 6 Conclusion

In summary, this study investigated the potential relationship between SD and GERD symptoms. We found that GERD patients with SD had a higher incidence of erosive esophagitis with more severe damage to the esophageal mucosa. Meanwhile, GERD patients with SD also had increased sympathetic activity while decreased parasympathetic or vagal activity, which indicated that autonomic function may be a potential pathophysiological mechanism.

## Data Availability

The original contributions presented in the study are included in the article/supplementary material; further inquiries can be directed to the corresponding authors.
